# Establishment and application of an intelligent management platform for home medication for children with leukemia

**DOI:** 10.1371/journal.pone.0320790

**Published:** 2025-04-01

**Authors:** Dahui Zhong, Yang Liu, Lin Mo, Li Zhang, Xuelan Shen, Qiumeng Xiang

**Affiliations:** 1 Endoscopy Center, Children’s Hospital of Chongqing Medical University, Chongqing, China; 2 Department of Nursing, Children’s Hospital of Chongqing Medical University, Chongqing, China; 3 Department of Out-patient, Children’s Hospital of Chongqing Medical University, Chongqing, China; 4 Department of Hematology and Oncology, Children’s Hospital of Chongqing Medical University, Chongqing, China; 5 You You Bao Bei Hospital, Chongqing, China; National Defense Medical Center, TAIWAN

## Abstract

**Objective:**

This study aimed to establish an intelligent management platform for home medication for children with leukemia and verify its efficacy in enhancing home medication compliance in children with leukemia using intelligent approaches.

**Method:**

Children with leukemia who were treated in a hospital in Chongqing, China, between January and April 2022 were enrolled. The user intentions and requirements for an intelligent management platform for home medication were analyzed for both the patient and their family, leading to the establishment of an intelligent management platform with multiple ports. Children with leukemia who were at the maintenance stage of the treatment during July-December 2023, were randomly divided into control and experimental groups. For medication management, hard copies of chemotherapy records and regular outpatient follow-up data were collected from both groups. For the experimental group, data were also acquired from the intelligent management platform. The self-efficacy in rational medication, rate of missed/incorrect administration, and unplanned admission rate of the two groups were assessed after three months of intervention. The usage efficiency of the platform was also evaluated in children and caregivers in the experimental group.

**Results:**

The intelligent management platform for home medication for children with leukemia comprised a patient-end WeChat applet, a medical staff-end WeChat applet, and a Web management end, with each applet having distinct functions. After three months of intervention, the self-efficacy score in rational medication of the experimental group was significantly higher than that of the control group (33.93 *vs.* 30.03, *P < * 0.05). Furthermore, the rate of missed or incorrect administration of the experimental group was significantly lower than that of the control group (10% *vs.* 26.76%, *P < * 0.05). The rates of unplanned admission of the two groups did not differ significantly (*P >  0.05*). Moreover, the overall satisfaction of the experimental group of children and caregivers with the proposed platform was 94.29%.

**Conclusion:**

The proposed management platform for home medication for children with leukemia was associated with increased self-efficacy in the rational medication of these children and a reduced missed/incorrect administration rate, thereby providing an effective approach for more accurate and safer home medication for these children.

## 1. Introduction

Childhood leukemia has a high incidence, together with high survival and recurrence rates [1–3] and requires prolonged treatment for 2 to 2.5 years [4]. The standard treatment approach includes a comprehensive regimen combining intravenous infusions and oral chemotherapy drugs. Oral administration of chemotherapy and preventive antibiotics is generally continued at home for 1.5 to 2 years, and adherence to the regimen has a direct effect on disease progression and prognosis [5]. It has been estimated that only 75% of children with leukemia show good home medication compliance, while 57.5% of these children receive incorrect or missed medication doses during home medication [6,7]. Children with poor medication compliance had recurrence rates 2.5 times higher than those with good compliance [8], significantly increased frequencies of unplanned administration and days, and a 283% increase in medical costs [9]. Current studies on home medication in children with leukemia have focused mainly on health education, SMS alerts, and medication management platforms [10–12] to improve home medication compliance. However, these studies have tended to have single functions and weak timeliness and, therefore, cannot achieve real-time assessment, monitoring, early warning, and intervention for patients and medical staff, preventing full collaboration between medical staff and patients.

This cross-sectional survey study investigated the requirements of a management platform for home medication in children with leukemia. In this study, a digital intelligent management platform was established for the assessment, monitoring, recording, early warning, and intervention of home medication in these children, based on the Theory of Planned Behavior [13]. Currently, this theory has been applied to the construction of various health intervention programs in the medical field and has a significant positive effect on the treatment outcomes and recovery rates of patients [14–16].

The objective of this study was to achieve real-time assessment, monitoring, early warning, and early intervention, as well as to effectively prevent or reduce the missed/incorrect administration of home medication in children with leukemia, thereby improving medication compliance and enhancing treatment outcomes and prognosis.

## 2. Materials and methods

### 2.1. Establishment of a home medication management platform for children with leukemia

#### 2.1.1. *Analysis of requirements for a home medication management platform for children with leukemia.*

This study utilized a convenience sampling method for the selection of all children with leukemia, together with their caregivers, who had received treatment at the Department of Hematology and Oncology of a hospital in Chongqing, China, from January–April 2022. A self-designed general information questionnaire and a usage requirement survey questionnaire were used in the design of the management platform. A total of 132 effective copies were collected. The average age of the included children was 6.72 ±  3.87 years, and acute lymphoblastic leukemia was the most common type, accounting for 118 cases (89.39%). Moreover, the average age of caregivers was 35.36 ±  8.23 years, and 70.45% of these were the children’s mothers. In addition, 26.50% of the children and caregivers had used home medication management platforms related to leukemia, such as general medication alert tools such as “Medicine Helper” and “Medication Assistant” and WeChat official accounts focused on health education. Furthermore, 90.90% of the children and their caregivers were willing to use an intelligent management platform, while 9.10% refused due to unfamiliarity with smartphones or perceived lack of necessity. The preferred platforms were WeChat applets (79.50%), mobile apps (64.40%), and Intelligent Medicine Box (13.60%). The functional requirements for a platform were ranked in order of importance as follows: medication records (100.00%), medication alerts (96.21%), missed administration warnings (93.94%), communications (90.91%), medication knowledge and consultation (88.64%), and medication lists (71.79%). Some caregivers utilized the online pharmaceutical ordering function (Table 1).

**Table 1:** Functional requirements for home medication management platform for children with leukemia.

Item	Very Necessary n (%)	Necessary n (%)	Generally Necessary n (%)	Rarely Necessary n (%)	Unnecessary n (%)	Demand Rate (%)
Medication alert	81 (61.36%)	46 (34.85%)	5 (3.79%)	0 (0.00%)	0 (0.00%)	96.21%
Missed administration warning	80 (60.61%)	44 (33.33%)	8 (6.06%)	0 (0.00%)	0 (0.00%)	93.94%
Medication record	76 (57.58%)	56 (42.42%)	0 (0.00%)	0 (0.00%)	0 (0.00%)	100.00%
Medication list	45 (34.09%)	50 (37.88%)	22 (16.67%)	5 (3.79%)	10 (7.58%)	71.79%
Medication knowledge	63 (47.73%)	54 (40.91%)	15 (11.36%)	0 (0.00%)	0 (0.00%)	88.64%
Medication consultation	55 (41.67%)	62 (46.97%)	15 (11.36%)	0 (0.00%)	0 (0.00%)	88.64%
Data statistics	48 (36.36%)	59 (44.70%)	5 (3.79%)	12 (9.09%)	8 (6.06%)	81.06%
Communications	64 (48.48%)	56 (42.42%)	12 (9.09%)	0 (0.00%)	0 (0.00%)	90.91%

#### 2.1.2. *Platform development team.*

The platform development team included 13 members with an average age of 37.39 ±  2.37 years. The leader was a nursing professor and the outpatient department head. Other members included three IT engineers, one deputy chief physician, two attending doctors, six senior nurses, and one senior pharmacist from the Department of Hematology and Oncology. Furthermore, two senior nurses were responsible for the literature review, two senior nurses for the survey of home management platform requirements for children with leukemia, and two senior nurses were responsible for the functional design of the platform and overall communication and coordination. The senior pharmacist reviewed the medication information, while the project leader and physicians were responsible for process review and quality control. The IT engineers developed and maintained the platform.

#### 2.1.3. *The framework of the home medication management platform for children with leukemia.*

**Fig 1:**
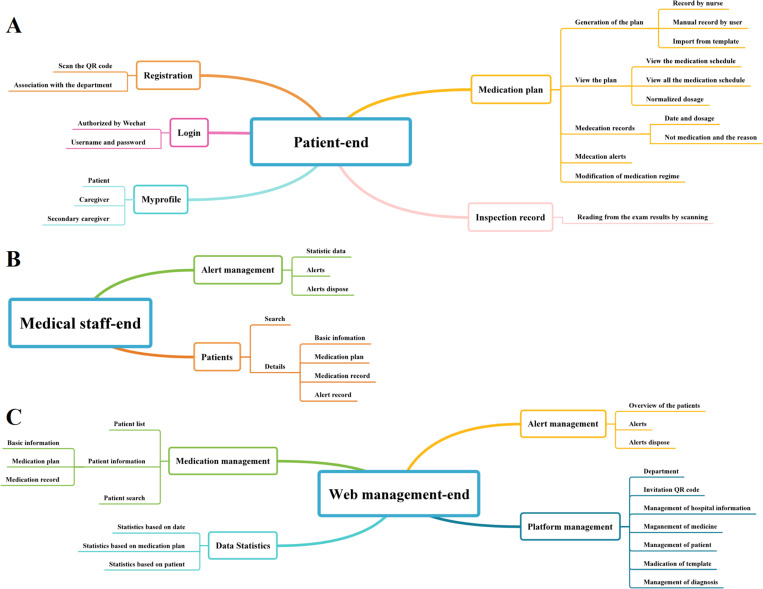
The frameworks of functional demands of different ends of the home medication management platform for children with leukemia. (A) The patient-end; (B) The medical staff-end; (C) The web management-end.

The functional framework of the platform was designed based on the preliminary survey of requirements, literature reading [10,17–19], discussion, and the Theory of Planned Behavior. The IT engineers with expertise in big data and intelligent engineering and two nurses communicated with each other to ensure that the platform functions were completely understood and finalized the functional framework, which included three main components, namely, the patient-end, medical staff-end, and Web management-end modules (Fig 1).

#### 2.1.4. *Construction of functional modules for the home medication management platform for children with leukemia.*

1) Patient-end WeChat applet

① Registration: The parents of the patients could access the platform through multiple channels. After completing the registration, individual patients could connect with multiple caregivers to share information, thus providing support for correct medication usage within the entire family. ② Medication plan: The caregiver of a child could choose an appropriate medication template based on the child’s current treatment stage, and set the dose, frequency and specific time according to the child’s medication requirements. Caregivers could modify the medication regimen based on the actual situation, as these patients often require dose and frequency adjustments, or may suspend medication based on their condition during home medication ③ Medication alerts: One hour before the medication time, a medication alert was pushed to the caregiver’s phone lock screen and the WeChat service manager. If the caregiver did not complete the medication record on time, another medication alert was sent one hour after the medication time. The patient’s WeChat applet medication alert page displayed basic information about the medication for that day, such as the medication time, dose, and breakdown (*e.g.,* 1/2 tablet). Due to the complex medication regimen for children with leukemia, which can vary in frequency, such as every other day, once a week, three days a week, and four days off, the medication alert page used different colored fonts to indicate which medications should be stopped the following day, reducing the chances of incorrect administration. ④ Medication records: The caregiver could select whether the medication had been taken or not. If taken, the dose consistency with the medication regimen was recorded. If the dose was inconsistent or the medication had not been taken, the caregiver was required to select the reason for the discrepancy, which could help medical staff understand the child’s medication situation at home and facilitate subsequent data analysis. ⑤ Examination record: Caregivers could upload test reports, such as complete blood, liver and kidney function. The platform would issue warnings for abnormal results, allowing medical staff to adjust the medication promptly according to changes in the patient’s physiological indicators (Fig 1A).

2) Medical staff-end WeChat applet

① Patients: The platform allowed the medical staff users to view basic information about the children and their caregivers, as well as the medication regime and alert records. ② Alert management: The platform displayed both medication alerts and other types of alerts (such as missed medication, incorrect dose, and incorrect medication type). Medical staff could then take appropriate interventional measures based on the alert information, such as manually pushing information or contacting by phone, with a recording of the handling process (Fig 1B).

3) Web management-end

① Information management: Authorization of administrator permissions super-administrator could input the basic information of staff from different campuses and departments, grant administrator permissions, and ensure the security of system usage. The input of information on medication and templates: Administrators could input various medications and basic inspection data, including medication names, specifications, images, and instructions, and establish various basic medication templates based on the chemotherapy regimen for the patients. ② Medication management: This allowed users to view the detailed medication records of the children (*e.g.,* medication time, dose, and frequency), medication alert records (*e.g.,* missed administration or incorrect dose), and the medication alert handling records of the medical staff. ③ Alert management: Medical personnel could implement relevant interventional actions based on the alerts and document the resolution. ④ Statistics: This allowed users to query basic information about all the children, and various statistics could be sorted in terms of medication period, medication regimen, and individual children, including the total number of doses, missed doses, taken doses, and medication alert counts. The reasons for missed or incorrect administration could be classified for a real-time analysis of the characteristics of poor medication compliance in these children (Fig 1C).

#### 2.1.5. *Platform trial operation and refinement.*

After the initial development, two specialized hematology and oncology nurses tested the platform’s stability, functionality, and ease of use and suggested improvements. The IT engineers made the necessary modifications. After safety tests and internal testing by the specialized nurses, 20 children and their caregivers were selected for a trial in the Department of Hematology and Oncology at our hospital. The IT engineers then made further modifications based on analysis of the collected feedback. Ultimately, the final version was established and launched (S1 and S2 Figs).

### 2.2. Application of the home medication management platform for children with leukemia

#### 2.2.1. *Subjects.*

This study included 148 children diagnosed with acute leukemia who received maintenance treatment at home following chemotherapy at a children’s hospital in Chongqing from July to December 2023, together with their caregivers.

Inclusion criteria:① Children aged between 1 - 18 years; ② Children who had been diagnosed with childhood leukemia based on the guidelines for childhood leukemia diagnosis and who were required to take home medication during the treatment period; ③ Children who were needed to take one or more forms of oral medication during the treatment period; ④ Children without severe mental illness or recent significant psychological stress. The inclusion criteria of caregivers included individuals who were 18‒60 years old, responsible for the child’s daily care for >  4 hours a day and were able to use the WeChat app.

Exclusion criteria: ① Caregivers who were unable to use smartphones, *i.e.,* the app and the applet; ② Children who boarded at school and were unable to use smartphones; ③ Children/caregivers who requested to withdraw during the study period.

Termination criteria: ① Cases where the interventional regimen was not implemented, or the entire treatment was not completed, thus affecting the efficacy analysis; ② Cases where the children’s physical condition deteriorated during treatment and must be terminated.

A formula for sample size estimation was used to compare two independent sample rates: When *α* =  0.05, the *Z* test was two-tailed with *Z*_0.05_ =  1.96, while the *β* test was one-tailed, and the power of the test was 0.9 with *Z*_*β*_ =  1.28. The sample size of the experimental and control groups was calculated to be 67, respectively. Allowing for a 10% follow-up loss, the final calculated sample size was *n*_1_ =  *n*_2_ =  74. Thus, a total of 148 children were grouped using a random number table into the experimental and control groups (*n* =  74/group). The first group was labeled A, and the second was labeled B. The 148 random numbers were sequentially placed in numbered, sealed, and opaque envelopes labeled from 1 to 148, and the envelopes were arranged in numerical order. The envelopes were distributed in the order of patient enrollment. Children who received envelopes marked A were assigned to the control group, and those who received envelopes marked B were assigned to the experimental group. Group allocation was concealed, and records were maintained by the project team members. If a child could not complete the study, this child and his/her caregiver were excluded from the study results.

#### 2.2.2. *Intervention method.*

Control group: Children with leukemia received conventional home medication management. Upon discharge, the doctor-in-charge filled out the child’s home medication records, including their basic information (name, age, gender, disease type), treatment stage, as well as medication dates, types, frequencies, and doses. The nurse in charge then provided the medications on discharge and explained the home medication regimen and precautions to the children and caregivers in person. After discharge, the children took medications at home and recorded on a paper form, i.e., whether the medication had been taken, as well as the dosage of the medication, among other information. Follow-up visits were scheduled once a month, during which the doctor reviewed the home medication record to understand the child’s medication status, addressed any questions about home medication, and adjusted the medication regimen based on changes in the child’s physiological indicators.

Experimental group: Other than the above measures applied in the control group, the intelligent platform for home medication also included: (1) Division of responsibilities in the research team; (2) Training of doctor-in-charge and nurses in the Department of Hematology and Oncology.

Consent and authorization were obtained from the director and head nurse of the Department of Hematology and Oncology. Then, training was organized for the doctor-in-charge and nurses involved. The training involved a demonstration of an explanation of the background, purpose, and significance of the platform application, demonstrating how to use the platform, assessing whether they had mastered the operation individually, and providing assistance to medical staff with login accounts and password setup. The doctors were responsible for controlling the accuracy of the medication regimen, handling adverse reactions during medication, and reviewing adjustments to the medication regimen. The nurses were responsible for handling medication alerts during home medication, answering questions related to the platform usage, handling platform malfunctions, and maintaining and upgrading the platform functions.

(3) Training of caregivers by the nurse-in-charge

Before patient discharge, the nurse-in-charge distributed the user manual of the WeChat applet to the caregivers, guided the caregivers in registration of the patient-end WeChat applet, and demonstrated how to create and modify a medication regime, record medication, handle alerts, view medication information, and query knowledge of the medication, accompanied by an assessment of their proficiency.

(4) Establishment of communication groups for caregivers and medical staff

A WeChat group was established for the research team for better coordination of various tasks. Furthermore, another WeChat group was established for caregivers and medical staff to communicate regularly. This allowed the caregivers to provide feedback on issues encountered while using the home medication management platform, enabling prompt response and resolution by the medical staff and IT engineers, ensuring smooth platform usage.

(5) Collaborative supervision of home medication through the intelligent management platform

The medication management platform sent a medication alert one hour before the due medication time based on the established medication regime. After taking the medication, the caregiver confirmed the dose in the WeChat applet. If the medication was not taken or the dose was incorrect, an appropriate reason was selected. In cases of missed medication, refusal to take medication, self-adjustment of the dose, or a wrong dose, the platform sent an alert to the WeChat applet of the medical staff. The nurse-in-charge checked the medication alerts every morning (07:30‒ 08:00), noon (12:00‒12:30), and evening (21:00‒22:00) and intervened *via* push notifications or phone calls. Furthermore, based on the child’s home medication status and changes in physiological indicators, the medical staff, patient, and caregiver jointly discussed and adjusted the medication regimen. Moreover, the medical staff regularly pushed knowledge related to disease treatment and chemotherapy drugs through the platform to improve awareness among both children and caregivers. The total duration of the intervention was three months.

#### 2.2.3. *Indicators.*

**Self-efficacy in rational medication** The Medication Event Monitoring System (SEAMS) [20] (with a Cronbach’s alpha coefficient of 0.934 and test-retest reliability of 0.932) was employed in this study. The scale comprises two dimensions and 13 items, including self-efficacy under uncertain conditions (5 items) and difficult conditions (8 items). Furthermore, it uses a 3-point Likert scale with scores of 1 to 3 for “not confident”, “somewhat confident”, and “very confident”, respectively. The total score ranged from 13 to 39, with higher scores indicating greater self-efficacy in adhering to medication.

**Missed/incorrect administration rate** The formula used for calculation was: missed/incorrect administration rate (%) =  number of children with missed/incorrect administration/total number of children taking medication ×  100%

**Unplanned admission rate:** The formula used for calculation was: unplanned admission rate (%) =  total number of unplanned admissions/total number of admissions ×  100%

**Performance of the intelligent management platform for home medication:** In this study, the scale developed by Peng *et al.* was employed to evaluate the efficacy of the intelligent medication management system. The scale has six dimensions, 15 items, and a content validity index (CVI) of 0.90, which indicates good content validity. Moreover, it uses a 5-point Likert scale from “strongly disagree” to “strongly agree”, with scores from 1 to 5, respectively. A higher score indicates higher satisfaction in the relevant dimensions.

#### 2.2.4. *Data collection.*

The data collection process was as follows: questionnaire preparation; approval from the head of the Department of Hematology and Oncology and the head nurses; unified training of the research personnel; the setting of appropriate schedules with the caregivers of the children; providing knowledge to the children and their caregivers about the purpose, significance, and method of the study; obtaining their consent for participation in the study; grouping of the included participants using a random number method. After three months of intervention, the survey personnel used paper questionnaires to assist the caregivers of the children in filling out the questionnaires one-on-one and face-to-face. The questionnaires were collected and immediately checked for completeness.

### 2.3. Statistical analysis

SPSS 24.0 was used for data analysis. Differences between the two groups were analyzed by independent sample *t*-tests. Count data are presented as percentages or frequencies, and the differences between the two groups were analyzed by the *χ*^2^ test and Mann-Whitney U rank-sum tests. *P < * 0.05 indicated statistically significant differences.

### 2.4. Ethics approval and consent to participate

This study was approved by the Ethics Committee of the CHCMU, China (2021 No. 308). All patients and caregivers provided written informed consent and volunteered to participate in the study when the general information was collected. All the data collected in thisthis study is for research use only, and all personal information was kept confidential. Allparticipants were assigned numbers for identification in the subsequent analysis.

## 3. Results

### 3.1. Characteristics of the two groups

A total of 148 children were enrolled in this study. However, in the experimental group, two subjects withdrew midway, and two changed treatment hospitals, resulting in a withdrawal rate of 5.4%. In the control group, two subjects lost contact, and one withdrew midway due to disease progression, resulting in a withdrawal rate of 4.1%. Therefore, 141 cases (70 in the experimental group and 71 in the control group) were included in the final analysis. The experimental and control groups did not differ significantly in terms of their basic information (*P* >  0.05) (Table 2). The self-efficacy scores in terms of rational medication in the two groups (30.40 ±  5.17 *vs.* 30.07 ±  3.31) indicated no statistically significant differences (*P* >  0.05).

**Table 2:** Basic information of the two groups.

Item	Subitem	Experimental group (n = 70)	Control Group (n = 71)	*χ*^2^/*z/t*	*P* value
Caregiver	Father	58 (82.86%)	49 (69.01%)	3.893	0.143
	Mother	9 (12.86%)	18 (25.35%)		
	The other	3 (4.29%)	4 (5.63%)		
Age of the caregiver (year)		32.06 ± 4.67	33.90 ± 7.36	-1.781	0.078
Education of the caregiver	Primary school	5 (7.14%)	7 (9.86%)	-1.49	0.136
	Junior middle school	19 (27.14%)	12 (16.90%)		
	Senior middle school	22 (31.43%)	15 (21.13%)		
	Junior college	7 (10.00%)	13 (18.31%)		
	Bachelor or above	17 (24.29%)	24 (33.80%)		
Occupation of the caregiver	Unemployed/freelance	24 (34.29%)	23 (32.39%)	5.661	0.129
	Farmer	12 (17.14%)	23 (32.39%)		
	Civil servants/employees of institutions	11 (15.71%)	11 (15.49%)		
	Business/self-employed	23 (32.86%)	14 (19.72%)		
Gender of pediatric	Male	35 (50.00%)	45 (63.38%)	2.571	0.109
	Female	35 (50.00%)	26 (36.62%)		
Age of pediatric (year)		4.73 ± 2.59	5.07 ± 3.08	-0.712	0.477
Pediatric in kindergarten or school	Yes	15 (21.43%)	15 (21.13%)	0.002	0.965
	No	55 (78.57%)	56 (78.87%)		
Annual household income (Yuan)		40000 (20000, 50000)	30000 (3000, 80000)	-1.591	0.112
Diagnosis of pediatric	Acute lymphoblastic leukemia	56 (80.00%)	57 (80.28%)	0.002	0.967
	acute non-lymphocytic leukemia	14 (20.00%)	14 (19.72%)		

### 3.2. Self-efficacy in rational medication in the two groups after intervention

**Fig 2:**
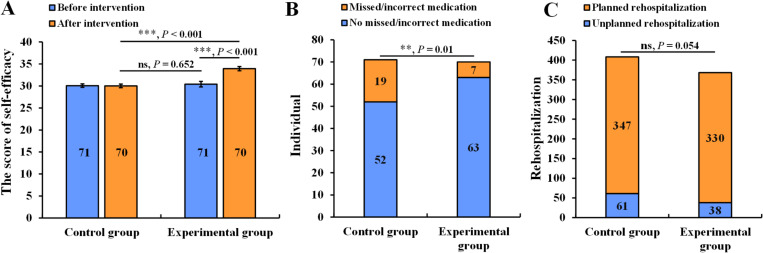
The effect of rational medication on self-efficacies (A), the missing/incorrect medication cases after intervention (B), and unplanned rehospitalization (C) in two groups. The difference in the effect of rational medication on self-efficacies was analyzed by independent samples *t* test, and the differences of the missing/incorrect administration cases after the intervention were analyzed by categorical data by *χ*^2^ test. The data was represented as means ±  standard error. The asterisks represent the significant differences, ** ****P**** <  0.01, *** ****P**** <  0.001. ns indicates no significant differences.

The self-efficacy score in rational medication of the experimental group three months after the intervention was significantly higher than that before the intervention (33.93 ±  3.93 *vs.* 30.40 ±  5.17, *P* <  0.001) (Fig 2A). Furthermore, the self-efficacy score of rational medication in the experimental group was significantly higher than that of the control group (33.93 ±  3.93 *vs.* 30.03 ±  3.40, *P* <  0.001).

### 3.3. Missing/incorrect medication in the two groups after intervention

The rate of missed/incorrect medication in the experimental group was 10% (7 cases), which was lower than that in the control group (26.76%), with a statistically significant difference (*P* =  0.01) (Fig 2B).

### 3.4. Unplanned rehospitalization in the two groups after intervention

The rate of unplanned rehospitalization in the experimental group was 10.33%, which was lower than that in the control group; however, the difference was not statistically significant (*P* =  0.054) (Fig 2C).

### 3.5. Evaluation of the medication management platform by children and caregivers

The satisfaction of children and caregivers in the experimental group with the usefulness, ease of use, information quality, and system quality of the medication management platform was >  95%, while the overall satisfaction with the platform was 94.29% (Table 3).

**Table 3:** The evaluation of the intelligent medication management system.

Item	Totally disagree n (%)	Disagree n (%)	Not sure n (%)	Agree n (%)	Totally agree n (%)	mean x *± * s	Satisfaction (%)
Usefulness							
It reminds me to take medication on time	0 (0.00)	0 (0.00)	0 (0.00)	7 (10.00)	63 (90.00)	4.90 ± 0.30	100
It improves my medication safety	0 (0.00)	0 (0.00)	0 (0.00)	6 (8.57)	64 (91.43)	4.91 ± 0.28	100
It increases the attention of medical staff to my child’s medication	0 (0.00)	0 (0.00)	0 (0.00)	0 (0.00)	70 (100.00)	5.00 ± 0.00	100
Ease of Use							
Learning to use it is easy for me	0 (0.00)	0 (0.00)	2 (2.86)	6 (8.57)	62 (88.57)	4.86 ± 0.43	97.14
Completing daily medication with it is simple	0 (0.00)	0 (0.00)	2 (2.86)	8 (11.43)	60 (85.71)	4.83 ± 0.45	97.14
I can use it proficiently	0 (0.00)	0 (0.00)	2 (2.86)	5 (7.14)	63 (90.00)	4.87 ± 0.41	97.14
Information Quality							
The information it provides is accurate	0 (0.00)	0 (0.00)	1 (1.43)	11 (15.71)	58 (82.86)	4.81 ± 0.43	98.57
The information it provides is sufficient	0 (0.00)	0 (0.00)	3 (4.29)	12 (17.14)	55 (78.57)	4.74 ± 0.53	95.71
It allows me to access required information in a timely manner	0 (0.00)	0 (0.00)	3 (4.29)	12 (17.14)	55 (78.57)	4.74 ± 0.53	95.71
System Quality							
It rarely malfunctions	0 (0.00)	0 (0.00)	0 (0.00)	8 (11.43)	62 (88.57)	4.89 ± 0.32	100
It responds very quickly	0 (0.00)	0 (0.00)	0 (0.00)	14 (20.00)	56 (80.00)	4.80 ± 0.40	100
Its user interface design is clear and reasonable	0 (0.00)	0 (0.00)	0 (0.00)	17 (24.29)	53 (75.71)	4.76 ± 0.43	100
Use Intention							
I want to continue using it in the future medication process	0 (0.00)	0 (0.00)	4 (5.71)	13 (18.57)	53 (75.71)	4.70 ± 0.57	94.29
I want to recommend it to fellow patients	0 (0.00)	0 (0.00)	8 (11.43)	13 (18.57)	49 (70.00)	4.59 ± 0.69	88.57
Even if it is charged, I am still willing to use it	0 (0.00)	11 (15.71)	4 (5.71)	28 (40.00)	27 (38.57)	4.01 ± 1.04	78.57
Overall Satisfaction							
Overall, I am satisfied with this system	0 (0.00)	0 (0.00)	4 (5.71)	6 (8.57)	60 (85.71)	4.80 ± 0.53	94.29

## 4. Discussion

Evaluation of the number and incidence of missed/incorrect administration after three months of intervention revealed a significantly reduced incidence of missed/incorrect administration in the experimental group compared with that in the control group (10% *vs.* 26.76%). This result indicated that compared with the conventional management of home medication in children with leukemia, the intelligent management platform for home medication with collaboration among medical staff, caregivers, and patients could effectively improve medication compliance and reduce the rate of missed/incorrect medication administration in these children. The results of this study are consistent with those of Psihogios and Walsh’s studies [11,21], which used information technology to assess the home medication management of children with leukemia. The platform proposed here provided data on the prescribed medication and had an automatic dose conversion function, assisting caregivers with low educational levels in learning about the medication. The platform provided repeated medication alerts and information-sharing among multiple caregivers, reducing medication compliance issues caused by unclear medication doses, travel, long absences from home, or changes in caregivers. Furthermore, this platform promoted collaboration between medical staff, caregivers, and patients and also enabled medical staff to monitor the home medication of children with leukemia in real-time, thus increasing the efficiency of home medication monitoring and management by the medical staff. It also allowed caregivers to record medication and report issues, reducing the spatial and temporal distance for communication with medical staff and thereby enabling timely feedback by medical staff and indirectly improving medication compliance in the children. The platform provided a statistical analysis of medication status and reasons for various alerts, enabling the medical staff to anticipate and identify high-risk children with poor medication compliance and take targeted intervention measures. It has been demonstrated that self-efficacy in rational medication plays an important role in improving medication compliance in patients with chronic diseases [22,23]. The application of this platform thus enhanced self-efficacy in rational medication and medication compliance.

Self-efficacy refers to an individual’s expectations, perceptions, confidence, or beliefs about their ability to successfully perform actions required to achieve specific goals. It is a driving factor in individual personality structure and plays an important role in individual psychological and behavioral development [24]. Here, it was found that the self-efficacy in the rational medication of the children was significantly higher after using the intelligent management platform for home medication compared with that in the control group before the intervention, consistent with the results of previous studies [19]. The proposed intelligent management platform for home medication obviates temporal and spatial limitations and allows communication between caregivers and medical staff in real-time, thereby providing prompt support and help from a professional medical team, especially important in cases of adverse reactions. Furthermore, with this platform, medical staff were able to participate in the home medication management of children with leukemia children and thus be more involved. Moreover, the platform allowed the association of multiple caregivers with an individual child, thereby enabling the sharing of information and joint supervision of the home medication, which helps prevent issues with medication compliance due to unfamiliarity with the medication regime when caregivers change. It has been found that the provision of information about rational medication to patients through the WeChat platform is more effective than traditional methods of medication education [25]. In this study, the medication management platform was linked to the official WeChat account of the Department of Hematology and Oncology at our hospital, which regularly pushed content on safe and standardized medication and its correlation with disease prognosis, chemotherapy medication precautions, and home care for children with leukemia. This strategy provides comprehensive health education for the children and can enhance their awareness of the importance of adhering to standardized medication. The use of the information platform, cooperation among family members, and timely professional support and intervention from medical staff can provide significant encouragement of both the children and their caregivers in the continuous and accurate adherence to the medication regime.

Studies have shown that children with poor medication adherence experience a 2.5 times higher rate of disease recurrence than those who adhere to the medication schedule. Moreover, both the frequency and duration of unplanned hospitalizations are markedly increased [8,9]. In the present study, the experimental group demonstrated a lower rate of unplanned readmissions than the control group; however, this difference was insignificant. It is important to note that unplanned rehospitalization rates are influenced by a variety of factors [26], with medication adherence at home representing only one component. Generally, children with leukemia undergo home medication treatment for approximately 1.5 years. The duration of the observation period in this study was shorter; therefore, the association between this platform and the incidence of unplanned readmissions among pediatric patients with leukemia requires additional verification.

The platform used in this study was comprehensive, allowing the recording of medication, reminders, risk alerts, caregiver-patient communication, real-time monitoring, intervention, and data statistics. The rate of satisfaction of the caregivers of the patients with this intelligent medication management platform was 94.29%, while the overall satisfaction with the usefulness, ease of use, information quality, and system quality was over 95%, demonstrating that both the leukemia patients and their caregivers had high acceptance of the platform, which met their needs for home medication management. These findings are consistent with patient evaluations of mobile healthcare platforms consisting of a web management console and an app [27]. Many recent studies have shown that software platforms designed and developed based on user needs are more scientifically sound and rational [22,28]. In the present study, the Theory of Planned Behavior-based medication management platform allowed a better understanding of the behavioral motivations and decision-making processes of pediatric leukemia patients and their caregivers. This, in turn, facilitated the design of a platform that aligned more closely with the needs of both the patients and their caregivers. The adherence of patients and caregivers to home medication regimens can be effectively improved by shaping the medication attitudes of pediatric leukemia patients, strengthening subjective norms, enhancing perceived behavioral control, reinforcing the intention to medicate at home, and promoting actual behaviors. Research has shown that interventional programs based on the Theory of Planned Behavior are effective in improving treatment outcomes and rehabilitation in patients [16]. However, the platform’s evaluation of medication adherence in pediatric patients relies primarily on self-reports from the patients and their caregivers on the platform, and thus, it lacks more objective evaluation indicators. Further, the Medication Event Monitoring System (MEMS) used to record the date/time when bottles are opened and closed assumes that patients take a specific dose at a particular time to assess medication adherence in pediatric patients [17]. Alternatively, the pill count method can be used, in which patients and caregivers are asked to return any remaining medication before the next prescription refill. Researchers can then determine the number of pills to be returned based on the current month’s prescription and dosage adjustments [29]. Medication boxes with radio frequency identification (RFID) tags can also be placed at drug-sensing terminals for swiping to identify the medications taken by patients and assess medication adherence [19]. These studies provide valuable references for objectively evaluating medication adherence in pediatric patients using this platform.

The platform is currently in continuous use among children with leukemia at a top-tier hospital and will also be applied to the management of home medication for other chronic diseases in children. The development and application of this platform can serve as a reference for the management of home medication in children with chronic diseases because the management process of home medication for different types of chronic diseases is similar. For regions without the WeChat app, its functionalities could also be integrated into other apps or developed into independent apps with the same framework. However, this study has some limitations. Due to time constraints, the intervention period in this study was relatively short, and the sample size was small. The long-term effects of the intervention, such as its impact on the rate of disease recurrence and quality of life, require further verification. Future efforts will focus on optimizing the platform’s functionalities and conducting large-scale, multi-center, high-quality studies to further verify its long-term effectiveness.

## 5. Conclusion

This study was based on the results of a previous survey on the need for an intelligent management platform for home medication for children with leukemia. The constructed intelligent medication management platform was found to improve the self-efficacy of both patients and caregivers in rational drug use and reduce the rate of missed or incorrect doses. The children and their caregivers showed high levels of acceptance and approval of the platform. Our future studies will extend the intervention period and increase the sample size to further optimize and upgrade the tool. Furthermore, the impact of its use on disease recurrence rates and the children’s quality of life will be analyzed to further verify its effectiveness.

## Supporting information

S1 Fig The screenshots of the patient-end applet for the home medication management platform for children with leukemia in the WeChat app. The personal information was hidden by a twill.
(TIF)

S2 Fig The screenshots of the medical staff-end applet for the home medication management platform for children with leukemia in the WeChat app. The personal information was hidden by a twill.
(TIF)
