# Supplementary figures and images for: Establishment and application of an intelligent management platform for home medication for children with leukemia
Source: PLoS One. 2025 Apr 1;20(4):e0320790. doi: 10.1371/journal.pone.0320790 (PMC11960947; doi:10.1371/journal.pone.0320790)

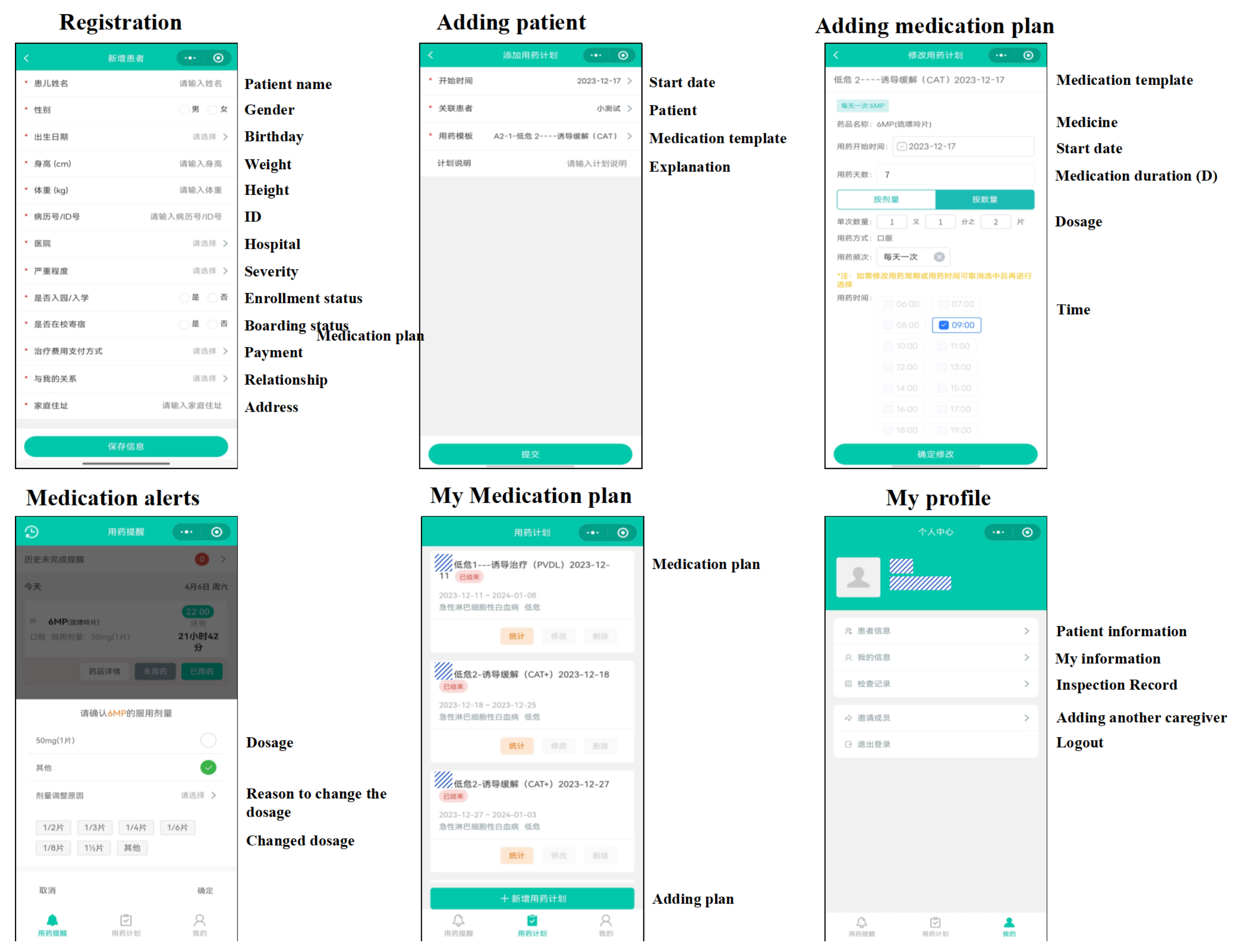

Supplement: S1 Fig — The personal information was hidden by a twill. (TIF) [file pone.0320790.s001.tif]

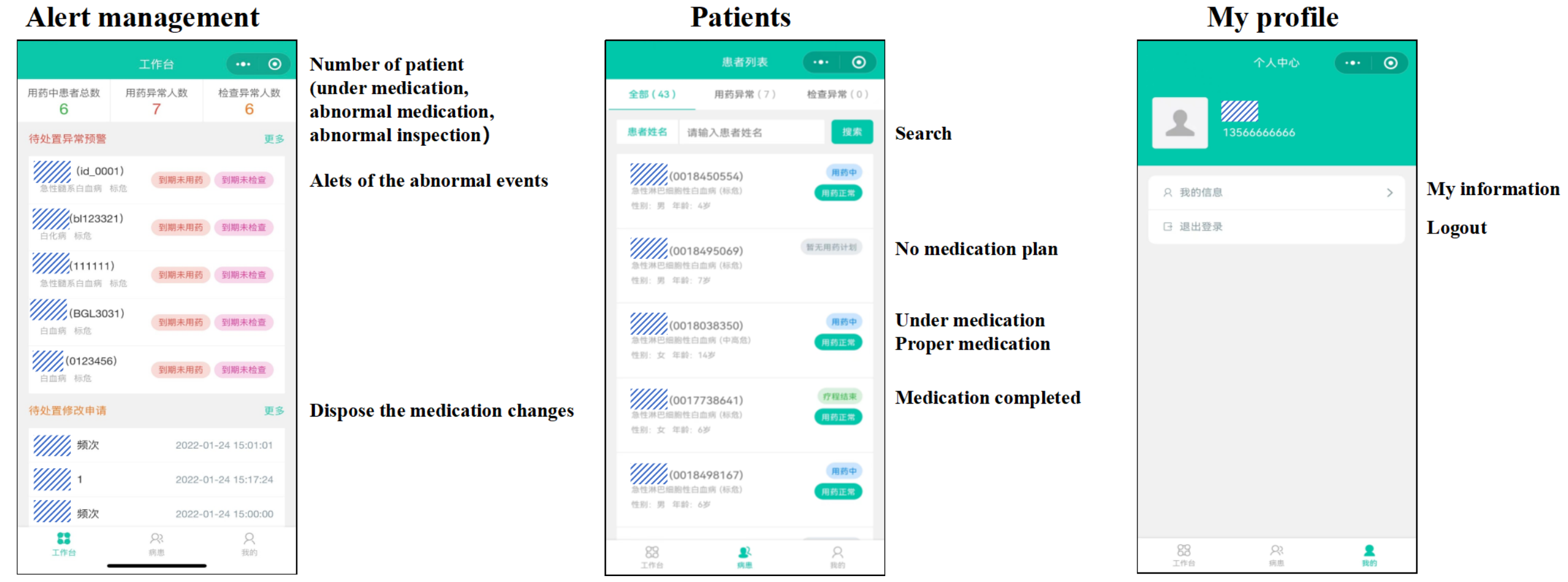

Supplement: S2 Fig — The personal information was hidden by a twill. (TIF) [file pone.0320790.s002.tif]
